# Evolution in the Management of Acute Myocardial Infarction in the Autonomous Community of Valencia (Spain): Ten Years of the Primvac Registry (1995-2004)

**Published:** 2010-06

**Authors:** Adolfo Cabadés, José Valencia, Joaquín Rueda, Ildefonso Echánove, Rafael Sanjuán, Javier Cebrián, Enrique González-Hernández, Juan Cardona, Francisco Colomina, Mercedes Francés, Victoria Ortolá, Francisco Sogorb

**Affiliations:** 1 *Unidad Coronaria, UCI, Hospital Universitario La Fe, Valencia, Spain;*; 2 *Servicio de Cardiología, Hospital General Universitario, Alicante, Spain;*; 3 *Servicio de Cardiología, Hospital Universitario La Fe, Valencia, Spain;*; 4 *Servicio de Cardiología, Hospital General Universitario, Valencia, Spain;*; 5 *Unidad Coronaria, UCI, Hospital Clínico Universitario, Valencia, Spain;*; 6 *Servicio de Medicina Intensiva, Hospital Universitario La Fe, Valencia, Spain;*; 7 *Servicio de Medicina Intensiva, Hospital de La Plana, Villarreal, Castellón, Spain;*; 8 *Servicio de Medicina Intensiva, Hospital de la Marina Alta, Denia, Alicante, Spain;*; 9 *Servicio de Medicina Intensiva, Hospital Universitario de San Juan, Alicante, Spain;*; 10 *Servicio de Medicina Intensiva, Hospital Arnau de Vilanova, Alicante, Spain;*; 11 *Servicio de Medicina Intensiva, Hospital General Universitario, Alicante, Spain*

**Keywords:** myocardial infarction, registry, treatment, mortality

## Abstract

**Introduction and objectives:**

Several registries of acute myocardial infarction (AMI) have been carried out in Spain, but few remain active. This work analyses the evolution of the characteristics and control of patients with AMI during the first 10 years of the PRIMVAC registry, initiated in 1995.

**Methods:**

The demographical and clinical characteristics, therapeutic-diagnostic procedures and pharmacological treatment of patients admitted with AMI between January 1995 and December 2004, were analysed in 17 coronary centres in the Autonomous Community of Valencia (South eastern Spain).

**Results:**

The mean age of the 19,719 patients recruited was of 65. The percentage of women, hypertension, hypercholestrolemia and diabetes increased during registry period. The median time of symptoms onset-hospital arrival was 151 minutes, without a decrease over the time, and the delay of thrombolysis fell from 200 to 154 minutes (*p*<0.01). Percentage of thrombolytic treatment oscillated between 39% and 48%. The mortality in the coronary units decreased (14.1% vs. 8.9%; *p*<0.001). The number of coronary angiography and percutaneous revascularisation performed increased up to 61% and 32%, respectively, of patients included. On discharge, the use of beta-blockers (29.3% vs. 66.7%), angiotensin-converting enzyme (ACE) inhibitors (41.7% vs. 57.9%) and statins (29.3% vs. 71%) went up.

**Conclusions:**

Overall mortality in the coronary unit decreased, without any variation in the incidence of serious complications. Time to thrombolysis was reduced over the time, with no significant increment in its use. The performance of coronary angiography and percutaneous revascularisation increased, with a low use of primary angioplasty. The use of beta-blockers, ACE inhibitors and statins increased at discharge.

## INTRODUCTION

Not long ago, the number of registries of ischemic heart disease in Spain was scant and knowledge about attendance data came in, mostly, from clinical trials. However, it is known that the information that these provide, often differ from what is obtained from the “real world” of the clinical attendance. During the last decade several registries, driven mainly by the scientific societies, have enabled us to know about the real situation of the ischemic cardiopathy in Spain (1-5). Nevertheless, the inclusion period is usually limited, and the registries that remain uninterrupted active for longer periods are not abundant. On the other hand, there exists a great variability in the clinical features, the risk factors, hospital treatment and mortality of the AMI in the different Spanish regions (2, 3). The registries put into practice and maintained in the Autonomous communities can be useful in the assessment scheme of the medical treatment of patients with AMI, with respect to the recommendations of the guidelines elaborated by the scientific societies and its evolution in time as well as for the comparison of the data obtained with other regional, national or international registries (6-9). The PRIMVAC registry (Project of Registry of Myocardial Infarction in Valencia, Alicante and Castellon), which was initiated on January 1995, has remained active uninterrupted, and it intends to obtain information on the medical attention of the patients with AMI in the Valencia Autonomous Community. The objective of this work is the description of the fundamental basic data of these patients, their management and its clinical evolution during the first 10 years of the PRIMVAC registry.

## METHODS

Just as it was mentioned in previously (1), all the hospitals with intensive care units or with coronary units (CU) of the Valencia Autonomous Community were invited to participate in the registry. 17 hospitals with health service coverage of the 70% of the Valencia Autonomous Community population were integrated in the PRIMVAC registry. The inclusion period was started in January 1995. Fourteen hospitals remained active until the end of the study. For the collection data, the UCIC computer programme (1), provided by the Ischemic Cardiopathy Section and the Coronary Units of the Spanish Society of Cardiology, was used. Data of each episode of AMI were sent to a coordination centre every three months, and there were investigators meetings four times a year, where collected data were analysed and updated. The variables used were previously defined (1, 10) and they included demographic features, risk factors, previous medical history of coronary disease, clinical complications, and diagnostic and therapeutic procedures. For the analysis of the medication prescribed at discharge a random sample stratified by hospitals of 25% of the global population included in the registry that were discharged alive, yearly from the CU of the participant hospitals. The diagnosis of the MI was made in accordance with the criteria of the World Health Organisation (11), and it required the presence of at least two of these three criteria: 1) Symptoms of AMI; 2) Elevated myocardial enzymes in blood; 3) characteristic electrocardiographic findings.

### Quality control

To evaluate the validity of the information of the PRIMVAC registry, a sample of 15% of the registered cases was analysed and an index of the kappa concordance was applied to the following variables: gender, previous medical history of diabetes, thrombolysis performance, highest killip class and MI location. In the case of age, coefficient of interclass correlation was applied. For all variables a concordance index higher than 70% was achieved. The analysis of exhaustiveness was carried out through the comparison of the contribution of cases with an independent source, the services of documentation and archives of the hospitals. The rate of exhaustiveness found of the PRIMVAC registry was 81.2% (1).

### Statistics

The unit of analysis was the health service episode. Continuous variables are presented as the mean ± standard deviation and the proportions as percentages. Categorical variables were analysed with the Chi-squared test with the correction of Yates when it was precise. In the case of reduced samples the exact test of Fisher was used. For the other quantitative variables the *t*-Student test was applied or not parametrical Mann-Witney approximation, in case the variable does not follow a normal distribution. Significant statistics for the tendencies was evaluated by means of the correlation of Spearman in the continuous variables, and with the Ji squared for the tendencies of Mantel in the categories. A value of *p*<0.05 (lineal tendency) was considered significant. The analysis was made with the statistical packets SPSS/PC+, S-Plus, Prism and PEPI.

## RESULTS

### Demographical data, risk factors and previous coronary disease

The PRIMVAC registry has gathered a total number of 19719 episodes of AMI, between January the 1^st^ 1995 and December the 31^st^ 2004. The demographical data, the proportion of cases with the diverse coronary risk factors, the previous medical profile and the ECG characteristics of the AMI index episode appear in Table 1. Median age is 65, without observing any significant variations during the 10 years of analysis. The percentage of women went up from 22.5% to a maximum of 26.6% (*p*=0.037). There was an increment in the percentage of patients with hypertension, hypercholesterolemia and diabetes, without any significant changes in either smoking or in the previous medical history of MI.

**Table 1:** Clinical variables of the patients included in the PRIMVAC registry

Year	Number of patients	Age	Female	Previous MI	Hypertension	DM	Smoking	Hyperchol-esterolemia

1995	2316	65 ± 12	22.5%	17.2%	42.7%	27.6%	37.3%	26.7%
1996	1768	65 ± 12	23.2%	16.9%	42.4%	27.3%	38%	26.7%
1997	1791	65 ± 12	24.2%	18.1%	47.7%	28.3%	36.9%	31.8%
1998	2073	65 ± 12	24.1%	17.1%	45.4%	28.4%	37.2%	29%
1999	2236	65 ± 12	24.8%	18.6%	46.6%	26.7%	36.8%	32.6%
2000	1886	65 ± 12	24.3%	17.7%	51.2%	29%	36.8%	32.4%
2001	2107	65 ± 12	26.6%	17.5%	51.2%	31.4%	35.1%	35.4%
2002	1936	65 ± 12	25.6%	16%	52%	32.3%	34.3%	33.5%
2003	1776	65 ± 13	24.3%	16.6%	51.7%	30.7%	37.1%	37.6%
2004	1830	65 ± 13	23.8%	17.1%	53.1%	32.7%	36.1%	37.7%
Total	19719	65 ± 12	24.3%	17.3%	48.3%	29.4%	36.5%	32.2%
*P*		0.51	0.037	0.41	<0.001	<0.001	0.065	<0.001

MI, myocardial infarction; HTA, Arterial hyper-tension; DM, Diabetes Mellitus.

### Electrocardiographic characteristics of the AMI

In Table 2, the ECG characteristics of the AMI are represented, observing a decrease of the Q wave AMI and an increment of the non Q AMI in the analysed period. The variations in the localisation of the AMI, though statistically significant, seem less relevant.

**Table 2:** Electrocardiographical characteristics of the patients analysed in the PRIMVAC registry

Admission year	Q wave present			AMI localisation			
	Yes	No	Not Precisable	Previous	Inferior	Mixed	Not precisable

1995	73.7%	20.8%	5.5%	42%	45.2%	1.6%	11.2%
1996	75.8%	18.6%	5.5%	43.9%	43.8%	2.3%	10%
1997	75.9%	19.8%	4.2%	41.1%	45.2%	3.5%	10.2%
1998	78.2%	16.7%	5%	42.8%	45.7%	2.9%	8.7%
1999	75.3%	19.9%	4.7%	42%	43.5%	2.3%	12.2%
2000	72.3%	24.2%	3.5%	41.4%	42.4%	2.3%	13.9%
2001	69.8%	26.4%	3.8%	40.7%	42.6%	1.7%	15%
2002	68.4%	26.9%	4.7%	43.1%	42.3%	1.2%	13.4%
2003	66.8%	28%	5.3%	43.2%	40.2%	1.7%	14.9%
2004	68.8%	26.2%	5%	41.1%	43.5%	1.4%	14.1%
Total	72.6%	22.7%	4.7%	42.1%	43.5%	2.1%	12.3%
*P*	0.001	0.001	0.001	0.001	0.001	0.001	0.001

### Thrombolysis

The percentage of thrombolysis in all the cases is situated in a band between 39% and 48%. It reached its maximum point in 1999, showing an ascending tendency from the beginning of the registry until 1999 and descending from the same year till the end of the inclusion period. If we consider only the cases of the Q wave-AMI, the percentage goes up from 48% in 1995 up to 61.7% in 2002 with a slightly decreasing trend afterwards. In Table 3, the use of the usual thrombolytics is observed.

**Table 3:** Percentage of patients treated with thrombolysis and thrombolytic used per year

	1995	1996	1997	1998	1999	2000	2001	2002	2003	2004	Total	*p*

Trombol1	39%	43%	46%	46%	48%	46%	45%	45%	39%	42%	44%	0.8
Trombol2	48%	50%	52%	54%	59%	58%	60%	61.7%	54%	58%	5.4%	0.01
rt-PA	57.4%	54.3%	58.4%	59.9%	56.6%	66.1%	60.4%	25.6%	18%	6.8%	47.7%	0.001
SK	35.3%	35.7%	29.7%	24.3%	24.1%	13.6%	14.4%	12%	9.2%	7.6%	21.1%	0.001
APSAC	6.7%	9%	10.6%	8.1%	4.9%	0.9%	0%	0%	0%	0%	4.2%	0.001
TNK	0%	0%	0%	0%	0%	0%	2.7%	46.9%	54.0%	66.2%	15.1%	0.001

Thrombol1, thrombolysis in all the patients; Thrombol2, thrombolysis in patients with Q wave infarction.

### Delay times

**Figure 1:**
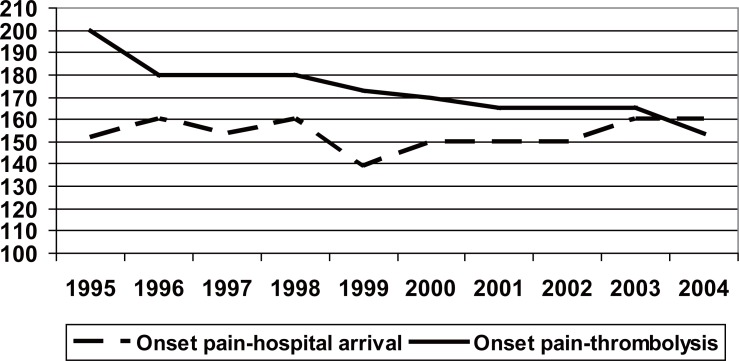
Evolution of the delay times during registry inclusion period. “pain-hospital” and “pain-thrombolysis”. The time-pain refers to all the cases of the PRIMVAC study and the time-pain thrombolysis refers only to those administered with a thrombolytic. *P*<0.01 statistical significance of the tendency.

The average interval between the onset symptoms and hospital arrival is 151 minutes, with a minimum value of 139 minutes in 1999 and a rise up to 160 minutes in 2003 and 2004, without any statistically significant decreasing trend. On the other hand, there exists a significant reduction in the delay until the thrombosis, which occurs at an average of 200 minutes in 1994 to 154 minutes in 2004 (Figure 1, *p*<0.001). The average time of arrival at the hospital, of the patients who underwent to thrombolysis was of 120 minutes (interquartile range of 70-122), was significantly less (*p*<0.05) than the average of the time of those not submitted to thrombolysis (240 minutes; intercuartil interval of 118-665).

### Medications used in the coronary units

There is a significant increase in the use of ACE inhibitors, betablockers, clopidogrel, Gp IIb/IIIa inhibitors, low molecular weight heparin and statins (Table 4). A diminution is observed in the use of unfractioned heparin.

**Table 4:** Medications used during the patient’s stay in the CU

	ASA	Clopidogrel	IIbIIIa blockers	Heparin	LWMH	ACE inhibitors	Betablockers	statins

1995	87.1%	NA	NA	53.1%	NA	27.0%	17.1%	NA
1996	84.2%	NA	NA	53.8%	NA	33.9%	16.0%	NA
1997	87.6%	NA	NA	61.4%	NA	39.0%	20.2%	NA
1998	90.1%	NA	NA	64.4%	NA	42.0%	21.7%	NA
1999	90.5%	NA	NA	68.2%	NA	43.1%	28.8%	NA
2000	89.1%	NA	8.5%	68.2%	36.6%	42.1%	28.8%	13.5%
2001	89.1%	8.7%	12.9%	59.9%	50.3%	44.6%	30.9%	26.0%
2002	91.6%	22.8%	14.7%	41.8%	64.9%	51.9%	36.1%	30.3%
2003	89.8%	35.0%	18.0%	29.3%	70.2%	51.5%	39.4%	41.3%
2004	93.0%	37.7%	19.6%	23.2%	69.8%	51.1%	41.8%	52.2%
Total	89.2%	24.3%	14.6%	53.0%	58.0%	42.3%	27.8%	32.2%
*p*	<0.001	<0.001	<0.001	<0.001	<0.001	<0.001	<0.001	<0.001

LWMH, low weight molecular heparins; NA, not available.

### Complications and mortality in the CU

There exists a significant diminution of the mortality in the CU, with figures that begin with 14.1% in 1995, reaching 8.9% in 2004 (Table 5). There are not any significant variations in the recurrence of MI (2.6%), stroke (0.8%) and cardiogenic shock (10.6%) during the years of study, but complete AV block and the atrial fibrillation incidence diminished.

**Table 5:** Mortality and clinical complications

Year	Death	Reinfarction	Stroke	Shock	Atrial fibrillation	Complete AV block

1995	14.1%	2.8%	1.2%	11.2%	11.0%	4.6%
1996	13.2%	2.2%	1.1%	10.9%	11.0%	6.7%
1997	13.3%	3.2%	0.5%	12.1%	9.5%	5.9%
1998	13.0%	2.9%	0.6%	11.1%	10.0%	5.6%
1999	13.0%	3.7%	0.7%	9.9%	9.2%	5.5%
2000	13.7%	2.2%	0.6%	12.7%	7.7%	5.5%
2001	10.7%	2.5%	0.5%	8.8%	8.1%	4.5%
2002	11.7%	1.8%	1.4%	9.9%	9.3%	3.5%
2003	10.1%	2.0%	0.9%	8.7%	8.7%	4.5%
2004	8.9%	3.0%	0.5%	11.0%	7.4%	4.3%
Total	12.2%	2.6%	0.8%	10.6%	9.2%	5.0%
*p*	<0.001	0.156	0.096	0.096	<0.001	0.001

### Diagnostic and therapeutic procedures

**Figure 2:**
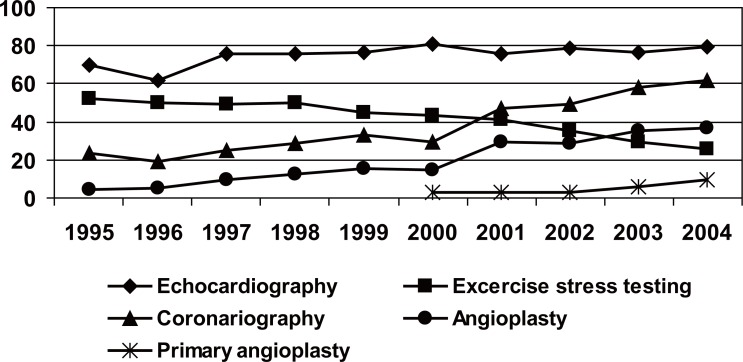
Diagnostic and therapeutic procedures carried out during the hospitalisation. For all variables *p*<0.01 statistical significance of the tendency.

The reduction in the use of the Swan-Ganz catheter and that of the provisional pacemaker (Table 6), is highlighted. In the analysis of the procedures carried out during the hospital stay (CU and the ward of Cardiology), summarised in Figure 2, there is an increment in the number of echocardiography and coronary angiography, reducing the percentage of exercise treadmill test before hospital discharge. The percentage of the patients submitted to percutaneous coronary revascularisation (PCI) rose from 5% in 1995 to 18% in 1999, reaching a 38% in 2004. The patients submitted to primary angioplasty were 2.6% in 2000 and 9.3% in 2004.

**Table 6:** Diagnostic and therapeutic procedures carried out in CU

	Provisional pacemaker	Cardioversion	Intra-aortic balloon counterpulsation	Mechanical Ventilation	Swan-Ganz catheter

1995	4.9%	4.5%	0.6%	8.2%	4.7%
1996	6.9%	4.1%	0.6%	8.0%	4.6%
1997	5.9%	2.8%	0.4%	6.6%	3.4%
1998	5.4%	2.8%	0.6%	7.4%	4.0%
1999	4.6%	3.4%	0.4%	7.3%	2.8%
2000	4.8%	3.4%	0.4%	8.5%	3.3%
2001	3.9%	3.4%	0.4%	7.9%	1.8%
2002	3.2%	3.4%	0.7%	7.0%	1.6%
2003	3.6%	2.8%	1.1%	6.5%	1.0%
2004	3.7%	2.5%	1.5%	6.5%	1.4%
Total	4.6%	3.3%	0.7%	7.4%	2.9%
*p*	<0.001	0.001	0.001	0.054	<0.001

### Treatment on hospital discharge

**Figure 3:**
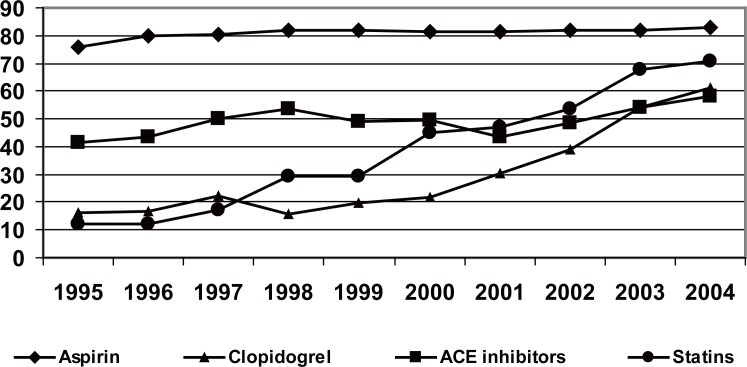
Harmacological treatment programmed for hospital discharge in percentages. For all variables *p*<0.01 statistical significance of the tendency.

**Figure 4:**
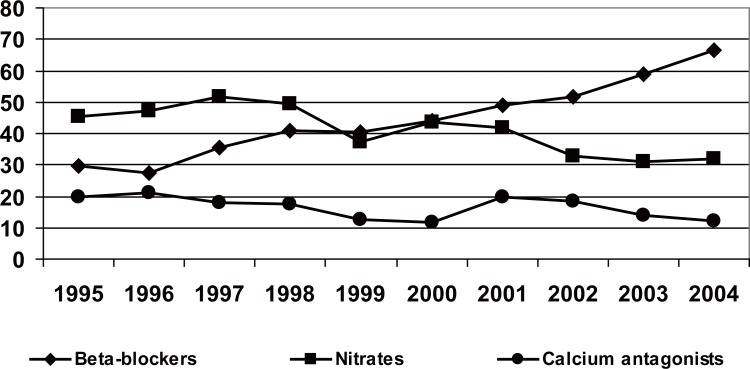
Pharmacological treatment programmed for hospital discharge in percentages. For all variables *p*<0.01 statistical significance of the tendency.

The administration of ASA was increased progressively until the year 1999, stabilising it further on, to around 86% (Figure 3). A very significant increment in the use of clopidogrel (57.9% in 2004) was observed, as well as in the usage of betablockers (66.7% against 29% in 1995), ACE inhibitors (57.9% against 41.7% in 1995) and statins (71% against 12% in 1994). On the contrary, the use of calcium antagonists and nitrates (Figure 4), fell in the last years.

## DISCUSSION

The PRIMVAC study is an active hospital registry, maintained without interruption, which has allowed us to know the evolution, in a period of 10 years, of the characteristics, prognostic features and management of the cases of AMI admitted to the CU of the 17 hospitals in the Valencia Autonomous Community, with a medical assistance coverage of 72% of the population of this Community. The quality control carried out during the study, has made it possible to check the validity of the information, as well as the rate of exhaustiveness. The analysis of treatments at hospital discharge has enabled us to obtain information about the evolution of the secondary prevention in the ten years of study. This information will be useful for the management comparison of patients with AMI between different periods in order to promote better strategies for the prognosis improvement of these patients.

### Demographic features, medical history and risk factors

The mean age in the PRIMVAC registry is similar to that of the Spanish registries, which include all the patients, without any age limit (2, 4, 5) and has not varied significantly for 10 years. The RISCI (4) registry indicates a little significant increment from 64.4% in 1995 to 65.2% in 1999. The proportion of women, which has increased from 22.5% in 1995 to 26.6% in 2001, is sensitively inferior to that of the other Spanish foreign registries (12-15), reached higher figures, to 30% and similar to the other Spanish registries (2, 4, 5). A statistically significant rise is observed in the medical histories of hypertension, hypercholesterolemia and diabetes with no modifications in the percentage of active smokers. These findings are also observed in the RISCI study (4), in the PRIAMHO II (5) and in the national registry of myocardial infarctions in the USA (NRMI) (16), even though smoking decreases in the latter. The increment of these risk factors in the population with AMI is difficult to explain. With respect to the failure of the preventive measures, another interpretation could be a better diagnosis of the risk factors in the general population in the last years.

### Thrombolysis

In the PRIMVAC study, the use of the thrombolysis is similar the one observed in other Spanish registries (2-5) and has increased in a progressive form, even though a stagnation and a tendency of descent, have been observed in the last years. A limitation to the comparison among registries is the fact that some of them include the AMI with and without Q wave. This may cause the real percentage of thrombolysis to be falsely low. Thus, a substudy of the PRIAMHO (17) shows that, the percentage of thrombolysis (42%) increases by ten points if only the cases with theoretical indication of thrombolysis (AMI with ST segment elevation), is considered. Thrombolysis is clearly justified in the AMI with ST elevation which, in the majority of cases, ends up developing Q wave. The use of thrombolytics in the PRIMVAC goes up, as far as 2002, where it reaches 61.7% of the AMI with Q wave. Though it falls slightly in 2003 and 2004, we need to bear in mind that in these years, primary angioplasty increases by 3 and 6 percentage points, for which the percentage of the revascularisation, has remained stable in the last years. The time up to the thrombolysis has diminished significantly throughout the ten years of the study. Given that the time between the beginning of the pain and the arrival at the hospital has not decreased, even noticing a change in the last years, it is possible that a better hospital planning of the emergency units with a reduction of the so called “door-to-needle time” can contribute to the diminution of the delay up to the thrombolysis.

### Complications and mortality

Mortality has decreased significantly throughout the time of the study, especially in the last two years, from 14.1% in 1995 to 8.90% in 2004. This tendency has also been observed in other registries (RISCI) (4) and has been pointed out in some studies like the PRIAMHO (18). The cardiogenic shock and the reinfarction, the two complications associated with a higher mortality following a AMI, have not varied in these years, while the use of beta blockers, ACE inhibitors and PCI have increased significantly. This enables us to venture into the hypothesis, already shown in other works (4, 18, 19), which the reduction of mortality is associated with a better treatment applied to those patients.

### Diagnostic and therapeutic procedures

The use of echocardiography increases up to 79% in 2004, a percentage similar to that of the most important Spanish registries (2-5). In the European registries, the use of this technique oscillates between 73% of the Euro heart survey ACS (12) and 89% of the French registry USIC (13). On the other hand, the exercise test previous to the hospital discharge falls as far as 25.8% of the patients, a similar result to the Euro Heart Survey ACS (12). This is explained by a more aggressive handling of the patient with infarction, just as the scientific Societies of Cardiology recommend (20, 21), increasing the coronary angiography from 23.3% in 1995 to 61.6% in 2004, in the PRIMVAC, similar values to the Spanish (3, 5) and European registries (14, 15). It is admitted that together with a more invasive strategy, the major determinants of the increment in coronary angiography use, are the availability of a haemodynamic laboratory in place and the execution of primary angioplasty. In the patients of the PRIMVAC, analysed in the year 2004, primary ACTP was carried out in only 9.3% and rescue angioplasty in 7.5% of patients. The percentage of the programmed ACTP (excluding the primary and rescue angioplasty), increased significantly, reaching 23% in the year 2004. These results, similar to those of other studies, seem to indicate that, in the PRIMVAC study, the frequency of the predischarged programmed angioplasty is similar to that of other registries, but the number of patients submitted to primary PCI, is clearly less than most of the registries analysed. This is explained by the adoption of an act of protocol common in the Valencia Autonomous Community, since the year 2000, where primary ACTP is only carried out as first option in a small number of suppositions and clearly this strategy should be increased dramatically.

### Pharmacological treatment after discharge

The use of ASA, beta-blockers and ACE inhibitors are applied as quality indicators in the handling of the patients with AMI. In the PRIMVAC registry, an increment in the usage of these medications has been observed. The administration of ASA has been stabilised in around 86%, similar to PRIAMHO II (84.3%), EUROASPIRE II (90%) and Euro Heart ACS (88.5%). On the other hand, a very significant increment in the use of clopidogrel has been verified. With respect to this, we need to point out that the percentage of patients with AMI without ST elevation, was nearly 30% in 2003 and that, the percutaneous revascularisation and stent implantation have increased in the last years.

The progressive increment in the use of beta-blockers and ACE inhibitors is similar to the one observed is Spanish registries, though its prescription is less than that of the European registries. In consonance with the recommendations of the guidelines, the use of statins has increased in the last years, reaching 71%, a figure superior than that of the PRIAMHO 2 (45%) and that of the NRMI-4 (66%).

## LIMITATIONS

In September 2000, the new definition of AMI, elaborated by the European Society of Cardiology and the American College of Cardiology came out. The investigators of the PRIMVAC study, which was already in its 6^th^ year of activity, after a wide discussion and bearing in mind that the fundamental objective of the registry was the comparison and analysis of tendencies, they decided to maintain the initial classical definition of AMI as the best option, at least from the epidemiological point of view.

The PRIMVAC study does not include the patients with AMI admitted directly to hospitalisation wards. These patients can mean a percentage higher than 10% of the total of the admitted patients with AMI and present different clinical and prognostic features. To obtain information on the diagnostic and therapeutic procedures carried out in the hospitalisation ward floors after the discharge of the CU, and about the treatment at the moment of the hospital discharge, only a sample of 25% of the patients was analysed. The logistic of the study did not permit the analysis of all of them. Nevertheless, this sample was at random and stratified by the hospitals, and it permits, from our point of view, a reasonable approach to the real data of the total number of patients.

## CONCLUSIONS

The contributed data show the usefulness of the PRIMVAC registry in the monitoring of the evolution changes in the diagnostic-therapeutic handling of the myocardial infarction, and permit an analysis of the grade of incorporation of the new clinical recommendations. Even though the incidence of severe complications of the AMI has not shown any important variations, the mortality in the CU has diminished in a relevant and significant manner, within the analysed period. The time up to the thrombolysis has decreased significantly, but it is still far from the recommended ideal time.

Among the diagnostic techniques, the use of the echocardiography is similar to that of the other analysed registries. Together with an increment in the coronary angiography, a progressive diminution of the execution of the exercise tests is observed. Though the percutaneous revascularisation has increased in a relevant form, the performance of primary angioplasty is low, obviously inferior to that of the communities and our neighbouring countries.

The use of ASA, beta-blockers, ACE inhibitors and statins, indicators of the quality of the handling of AMI, has increased in a relevant and significant manner and it is similar to that of other Spanish registries. An increment in the use of clopidogrel and a progressive fall in the prescription of calcium antagonists and nitrates are confirmed on hospital discharge.
